# Factors Associated with Nodal Upstaging in Clinical T1a-bN0M0 Non-Small Cell Lung Cancers

**DOI:** 10.3390/cancers14051277

**Published:** 2022-03-01

**Authors:** Tung-Ming Tsai, Chao-Yu Liu, Mong-Wei Lin, Hsao-Hsun Hsu, Jin-Shing Chen

**Affiliations:** 1 Department of Surgery, National Taiwan University Hospital and National Taiwan University College of Medicine, Taipei 10002, Taiwan; 019881@ntuh.gov.tw (T.-M.T.); mwlin@ntu.edu.tw (M.-W.L.); ntuhsu@gmail.com (H.-H.H.); 2 Department of Surgical Oncology, National Taiwan University Cancer Center, Taipei 106037, Taiwan; 3 Department of Surgery, Far Eastern Memorial Hospital, New Taipei City 220216, Taiwan; chaoyuliu@hotmail.com

**Keywords:** nodal upstaging, non-small cell lung cancer (NSCLC), video-assisted thoracoscopic surgery (VATS), lobectomy, consolidation–tumor (C/T) ratio

## Abstract

**Simple Summary:**

The incidence rate of lymph nodal upstaging after curative surgical resection is approximately 10% in clinical stage I non-small cell lung cancer (NSCLC), which can significantly affect the prognosis. The aim of our retrospective study was to reassess the predictive factors of nodal upstaging in patients with clinical T1a-bN0M0 adenocarcinoma. In a cohort of 352 patients with clinical T1a-bN0M0 adenocarcinoma who received standard lobectomy and lymph nodal dissection, 28 (7.95%) patients had lymph nodal upstaging. The significant risk factors include abnormal serum carcinoembryonic antigen levels, solid part tumor diameter ≥ 1.3 cm, and consolidation–tumor ratio ≥ 0.50 on chest computed tomography. Standard lobectomy is recommended for patients with these predictive factors. If neither of the predictive factors are positive, a less invasive procedure may be a reasonable alternative. Further studies are needed to verify these data.

**Abstract:**

Nodal upstaging of lung adenocarcinoma occurs when unexpected pathological lymph node metastasis is found after surgical intervention, and may be associated with a worse prognosis. In this study, we aimed to determine the predictive factors of nodal upstaging in cT1a-bN0M0 primary lung adenocarcinoma. We retrospectively reviewed a prospective database (January 2011 to May 2017) at National Taiwan University Hospital and identified patients with cT1a-bN0M0 (solid part tumor diameter ≤ 2 cm) lung adenocarcinoma who underwent video-assisted thoracoscopic lobectomy. Logistic regression models and survival analysis were used to examine and compare the predictive factors of nodal upstaging. A total of 352 patients were included. Among them, 28 (7.8%) patients had nodal upstaging. Abnormal preoperative serum carcinoembryonic antigen (CEA) levels, solid part tumor diameter ≥ 1.3 cm, and consolidation–tumor (C/T) ratio ≥ 0.50 on chest computed tomography (CT) were significant predictive factors associated with nodal upstaging, and patients with nodal upstaging tended to have worse survival. Standard lobectomy is recommended for patients with these predictive factors. If neither of the predictive factors are positive, a less invasive procedure may be a reasonable alternative. Further studies are needed to verify these data.

## 1. Introduction

Non-small cell lung cancer (NSCLC) with small tumor size is now frequently detected due to the prevalence of computed tomography (CT) as a screening tool for pulmonary lesions. Although lobectomy with systemic lymph node dissection (LND) is considered the standard treatment for early stage NSCLC [1,2], there is still controversy on how to harvest the lymph nodes and determine the extent of LND, leading to the debate on surgical management choices.

Precise clinical staging, especially in determining the lymph nodal status, is essential before deciding on the surgical procedure for curative surgery. Although preoperative invasive surgery (mediastinoscopy or endobronchial ultrasound biopsy) is the gold standard for detecting lymph nodal metastasis, it is controversial in cases with small pulmonary nodules or the absence of suspicious lymph nodes on preoperative imaging [3]. Lymph nodal upstaging, also known as occult metastasis, can significantly affect the prognosis, especially when there is mediastinal or contralateral involvement. The incidence rate of lymph nodal upstaging is approximately 10% in patients with clinical stage I NSCLC [4,5]. Several clinical risk factors are associated with this unfavorable situation.

Nodal upstaging occurs when unexpected pathological lymph node involvement is found in the final evaluation of surgical specimens and is not infrequent in early stage NSCLC [5,6,7,8]. When compared to other histopathological cell types, adenocarcinoma comprises the largest number of cases of NSCLC [9,10], and is mostly prone to nodal upstaging in cT1N0 disease [10,11,12]. In addition, tumor size is associated with nodal upstaging, even in T1 diseases. Patients with tumor size between 2 and 3 cm showed more frequent nodal upstaging compared to tumor size of 2 cm or less [13,14,15].

With the growing number of early-stage lung cancer detections, accurate prediction of lymph node disease would be crucial in deciding on the surgical strategy. In this study, we aimed to reassess the predictive factors of nodal upstaging in patients with T1a-bN0M0 adenocarcinoma.

## 2. Material and Methods

### 2.1. Study Population

We retrospectively reviewed 2148 patients with primary NSCLC who underwent surgical tumor resection by a single surgical team at the National Taiwan University Hospital from January 2011 to May 2017. The data used in the analysis were extracted from a prospectively collected database, where all patients were treated by the same surgical team using the same protocols, including preoperative evaluations, surgical management, postoperative care patterns, and clinical follow-ups. The Research Ethics Committee of National Taiwan University Hospital approved this study (approval number 201803010RINB; 28 January 2019), and the requirement for informed consent was waived due to its retrospective nature.

**Figure 1:**
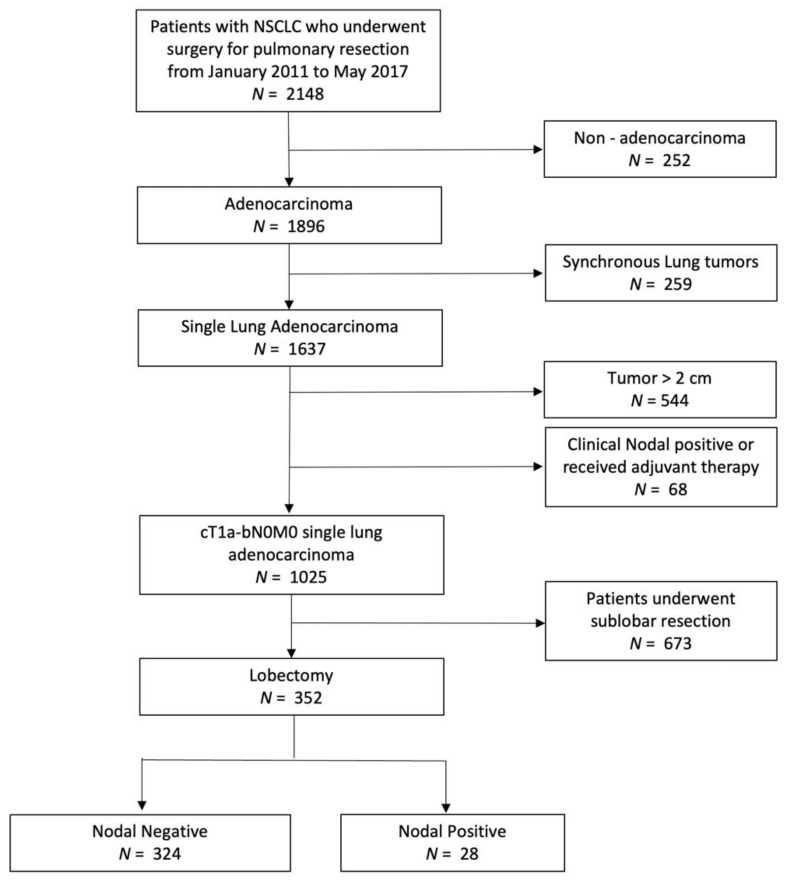
Algorithm for patient selection. NSCLC non-small cell lung cancer.

Patients included in this study had a clinical diagnosis of cT1a-bN0M0 adenocarcinoma, and then underwent curative lobectomy and complete mediastinal lymph node dissection. Patients were excluded if they had non-adenocarcinoma (*n* = 252), synchronous multiple adenocarcinoma (*n* = 259), clinical tumor size > 2 cm (*n* = 544), clinical evidence of positive lymph nodal or distant metastasis, neoadjuvant treatment (*n* = 68), or sublobar resection (*n* = 673). Figure 1 illustrates the patient selection algorithm. Finally, 352 patients were included in the study.

### 2.2. Preoperative Assessments

All patients underwent preoperative staging workups within 2 months before the surgery, which included chest and abdominal CT, brain CT or magnetic resonance imaging (MRI) of the brain, bone scintigraphy or positron emission tomography (PET), serum carcinoembryonic antigen (CEA), and lung function test. 

In the preoperative assessments, we used contrast-enhanced chest CT scans to evaluate the entire thoracic cavity. A commercially available viewer (IMPAX 5.2; Agfa HealthCare N.V., Mortsel, Belgium) was used for the CT imaging measurements. Four experienced thoracic surgeons (TMT, XHC, HCL, and MWL) measured and recorded the size of consolidation, ground-glass opacity (GGO) component, and total tumor size for every patient using the lung window level setting. The consolidation–tumor (C/T) ratio, which was defined as the ratio of the maximum diameter of consolidation to the maximum diameter of the tumor according to JCOG0201 [16], was used to describe the local invasiveness of the primary lung tumor. Tumors with pure ground-glass opacity and no solid component had a C/T ratio of 0. For pure solid tumors, the consolidation size was equal to the total tumor size, and the C/T ratio was 1.

Preoperative lymph node status was assessed using CT or PET/CT. Lymph nodes with either diameter ≥ 10 mm in the short axis or nodal fluorodeoxyglucose uptake with SUVmax ≥ 2.5 were considered clinically positive. Further assessments of positive lymph nodal metastasis included endobronchial ultrasound-guided needle biopsy, mediastinoscopic biopsy, or thoracoscopic surgical biopsy, if necessary. The central location of the tumor was defined as the inner one-third of the hemithorax.

### 2.3. Surgical Techniques and Follow-Ups

All procedures were performed using video-assisted thoracoscopic surgery (VATS). Before each lobectomy, a definitive diagnosis of lung cancer must first be obtained. For a centralized tumor, preoperative endobronchial ultrasound biopsy or percutaneous CT-guided biopsy was arranged. For some peripheral indeterminate pulmonary nodules, a diagnostic wedge resection followed by a frozen section was performed to confirm the diagnosis before the completion of lobectomy. Every lobectomy was performed with radical hilar and mediastinal lymph node exploration and dissection, which included peri-bronchial/hilar N1 stations (groups of 10, 11, 12) and mediastinal N2 stations (groups of 3, 4, 7, and 9 for right-side tumors, and groups of 5, 6, 7, and 9 for left-sided tumors). All specimens were formalin-fixed and paraffin-embedded for microscopic examination. Clinical and pathological staging was evaluated according to the 8th edition of the American Joint Committee on Cancer (AJCC) TNM staging system [17]. Histopathological patterns were classified according to the 2015 World Health Organization criteria [18].

A PET-CT was performed for patients of high risk to detect any residual malignancies within 2 months after the surgery. At the same time, all patients were regularly evaluated in the outpatient clinic with physical examinations, serum CEA tests, and chest CT every 4–6 months, according to the physician’s instructions. Brain MRI or CT, PET or bone scan, bronchoscopy, chest or endobronchial ultrasound, and other tests were performed whenever any suspect tumor recurrence were noted. Diagnostic biopsies, such as thoracentesis, needle biopsy or surgical biopsy, were further performed for tissue proof cytologically or histologically if necessary.

### 2.4. Statistical Analysis

We recorded the demographic and clinical data from chart reviews for each patient, including age, sex, smoking history, familial history of lung cancer, preoperative CEA level, tumor size and location, surgical approach, and intraoperative findings. Histopathological type, dissected number, and lymph node status were also recorded from the pathology reports. 

Continuous variables are presented as mean ± standard deviation, and the two-sample *t*-test or Mann–Whitney U-test was used for statistical analysis. Categorical variables were presented as frequency (percentage), and the Pearson’s chi-square test or Fisher’s exact test was used for statistical analysis. All data analyses were carried out using SPSS version 25 (SPSS; IBM, Chicago, IL, USA), with statistical significance set at *p* < 0.05. 

Overall survival and disease-free survival rates were analyzed using the Kaplan–Meier method and log-rank test. Multivariate analysis was performed using the Cox regression model, which adjusted for significant confounding factors in the univariate model. Receiver operating characteristic (ROC) curve analysis was performed to assess the predictive ability of occult lymph nodal metastasis. A cut-off point reflects sufficiently high sensitivity and specificity for distinguishing nodal metastases.

## 3. Results

### 3.1. Patient Demographics and Radiological Findings

In this study, we retrospectively reviewed 352 patients with clinical T1a-bN0M0 lung adenocarcinoma who underwent thoracoscopic lobectomy and radical hilar and mediastinal lymph node dissection. Females (*n* = 228, 64.8%) and non-smokers (*n* = 301, 85.5%) comprised the majority of this cohort. Most of the tumors were located in the right upper lung lobe (*n* = 133, 37.8%) and positioned relatively peripherally, while approximately 1/4 of the tumors were more centrally positioned (*n* = 77, 23.7%). Among them, 28 patients were found to have histopathological lymph nodal metastasis (pN+ group), while the other 324 patients did not (pN− group). The overall nodal upstaging rate was 7.95%. There was no difference in age, sex, smoking status, familial history, or other underlying malignancies between these two groups. Table 1 shows the clinicopathological characteristics of these two groups.

The main differences between these two groups arose from the clinical radiological features, including tumor size, C/T ratio, and tumor location. The overall tumor size was significantly different (median total tumor diameter, pN+: 2.10 cm; pN−: 1.93 cm, *p* = 0.049) between the two groups. Patients in the pN+ group had a significantly larger solid component (median = 1.60 cm, interquartile range (IQR) = 1.40–1.81 cm) than the patients of the pN− group (median = 0.85 cm, IQR = 0–1.38 cm, *p* < 0.001). Patients in the pN+ group had a significantly higher C/T ratio (median = 0.75, IQR = 0.59–0.87) than patients in the pN− group (median = 0.43, IQR = 0–0.66, *p* < 0.001). For a subgroup of GGO predominant tumors (C/T ratio < 0.5, *n* = 200), the solid component was significantly larger in the pN+ group (*n* = 3, median = 1.45 cm) when compared with the pN− group (*n* = 197, median = 0.32 cm, *p* = 0.006). The location of the tumor was also different. Tumors in the pN+ group tended to be located near the pulmonary hilum (46.4% vs. 19.8%, *p* = 0.003). Another clinical difference was the preoperative tumor marker. Although patients of both groups had average CEA levels within a normal range (pN+ group: median = 2.04, IQR = 1.28–5.67 ng/mL; pN− group: median = 1.54 ng/mL, IQR = 1.42–2.48 ng/mL), patients in the pN+ group had a significant higher probability of abnormal CEA (*n* = 6, 21.7%) than patients in the pN− group (*n* = 12, 3.7%, *p* = 0.001). In the analysis of 18 cases of CEA alterations, seven cases (38.9%) had CEA < 5 ng/mL within 6 months after surgery. The median CEA level preoperatively was 10.1 (IQR: 8.15–21.75), and the median CEA level postoperatively was 5.67 (IQR: 2.27–18.49). In a subgroup analysis of non-smokers (*n* = 301, pN+: 24, pN−: 277), CEA alteration was significantly higher in pN+ (pN+: 25% versus pN−: 3.25%, *p* < 0.001).

### 3.2. Histopathological Findings

The pathological tumor size in the pN+ group (median = 2.2 cm, IQR = 1.73–2.5 cm) was significantly larger than in the pN− group (median = 1.6 cm, IQR = 1.2–2.1 cm, *p* < 0.001). The number of examined lymph nodes was similar between the two groups (median = 13.5 in pN+ vs. median = 13 in pN−, *p* = 0.852). In the pN+ group, the median number of positive lymph nodes was 2 (IQR = 1–3), one in each of N1 (IQR = 0–2) and N2 (IQR = 0–2.75). In the pN+ group, 60.7% (17/28) of the patients had occult N1 involvement, 57.1% (16/28) of the patients had occult N2 metastasis, and 25% (7/28) of the patients had both metastatic N1 and N2 lymph nodes. Considerable differentiation of the tumor was only found in the pN− group (*n* = 107, 33%), and lymphovascular invasion was more frequent in the pN+ group (71.4% vs. 8%, *p* < 0.001). Table 2 showed the results of somatic mutation. The somatic genetic study was applied in about one-third in our cohort (124 cases of EGFR; 109 cases of BRAF, KRAS and HER2; 42 cases of ALK). The EGFR mutation was most common (pN+: *n* = 4, 44.4%; pN−: *n* = 79, 68.7%, *p* = 0.155). In the pN+ group, only mutations of EGFR and ALK were found. However, there was no significant difference of genetic mutation between these two groups.

### 3.3. Selecting Cut-Off Values to Discriminate Patients with Lymph Nodal Upstaging

Three continuous variables were used to predict lymph nodal upstaging, including total tumor diameter, solid component diameter, and C/T ratio. All tumors in the pN+ group measured at least 1 cm in the solid component in clinical staging (range = 1.07–2.00 cm). With regard to the cut-off value obtained by the ROC curve, the cut-off diameter of the solid component that had the best combined sensitivity and specificity was 1.3 cm (area under the curve (AUC) = 0.797; *p* < 0.001). The cut-off value of the C/T ratio for predicting lymph nodal upstaging was 0.579 (AUC = 0.755; *p* < 0.001), and the cut-off value of the total tumor size was 1.74 cm (AUC = 0.610, *p* = 0.049). 

### 3.4. Univariate and Multivariate Analyses for Predictors of Lymph Nodal Upstaging 

Table 3 shows the risk factors associated with lymph nodal upstaging according to logistic regression analysis. In univariate analysis, the CEA level, diameter of the solid part of the tumor, C/T ratio, and tumor location had *p* values less than 0.05; therefore, these factors were included in the multivariate model. Multivariate analysis of the four risk factors obtained from univariate analysis demonstrated that serum CEA level (≥5 ng/dL vs. <5 ng/dL, odds ratio (OR = 6.80, 95% confidence interval (CI) 1.89–24.40, *p* = 0.03), solid part diameter (≥1.3 cm vs. <1.3 cm, OR = 4.36, 95% CI 1.39–13.68, *p* = 0.012), C/T ratio (≥0.50 vs. <0.50, OR = 4.93, 95% CI 1.16–21.02, *p* = 0.031), and tumor location (central vs. peripheral location, OR = 4.64, 95% CI 1.84–11.70, *p* = 0.001) were independent and significant risk factors for nodal upstaging. Although the C/T ratio ≥ 0.75 had a significant difference in univariate analysis, there was no significant difference in multivariate analysis. In univariate analysis, abnormal CEA was a significant predictor of lymph nodal upstaging (OR = 9.926, 95% CI = 3.18–30.97).

### 3.5. Correlation between Nodal Upstaging and Survival

In our study cohort, the median follow-up period was 64 months (IOR = 48–83 months). During this period, we lost follow-up in two cases, and a total of 11 cases passed away. When compared to the pN+ group, the patients in the pN− group had a significantly greater 5 year overall survival and 5 year disease-free survival (98.5% versus 75.9%; 90.3% versus 51.5%, respectively). 

## 4. Discussion

In the present study, we identified that the larger size of the solid component, C/T ratio, tumor location, and CEA level were significant predictors of occult lymph nodal metastasis in clinical T1a-bN0M0 NSCLC patients.

The larger the tumor, the higher the chance of invasion of the lymphatic vessels and the greater the chance of spreading. However, the T component of the AJCC 8th edition staging system is described on the basis of solid or invasive size alone. Tumor size is determined by the largest dimension of the solid part by CT or the pathological invasive part. The size of the non-invasive part, also called the GGO pattern, or the lepidic component, was not considered to have an impact on survival. Furthermore, recent studies demonstrated that partly solid tumors, especially GGO-dominant tumors, are likely to be localized and indolent, which would be adequate for nodal sampling, or may not require any nodal dissection in selected cases.

However, there is growing evidence that GGO components are associated with prognosis in part-solid GGOs. Part-solid tumors may have a better prognosis than pure-solid tumors in clinical stage IA patients, according to the TNM classification (8th edition), and similar results were found for the T1a-1b (≤2 cm) subgroup [19]. However, for tumors larger than 2 cm, there is not enough evidence to show that the GGO component has a significant effect on survival [20]. 

The Japan Clinical Oncology Group (JCOG) 0201 [21], a prospective study, suggested that the cut-off value of a C/T ratio could be classified into three levels: <0.25, <0.5, >0.5. A non-invasive adenocarcinoma could be defined as an adenocarcinoma ≤ 2 cm with a C/T ratio ≤ 0.25. In our study, a C/T ratio ≥ 0.5 was used as a cut-off value for pathological noninvasiveness in part-solid GGO lesions, implying potential lymph nodal metastasis. This cutoff value has also been adopted in several studies [20,22,23]. Therefore, a C/T ratio may help determine the prognosis or treatment policy of part-solid GGO lesions. In contrast, considering only the size of the invasive component, a tumor size of more than 30 mm increased the possibility of nodal metastasis. In our study, we found no nodal upstaging in cases with nodular invasive parts less than 1 cm, which is similar to the findings of another study [8].

The relationship between the central location of a tumor and the increasing risk of lymph nodal metastasis was first described in 2006 [24]. However, there is a discrepancy in the definition of “central” location. The European Society of Thoracic Surgeons [25] defines ‘central’ tumors as being located within the inner two-thirds of the hemithorax. However, in our study, we used the same definition from the American College of Chest Physicians [26], which defines a ‘central’ tumor as being located in the inner one-third of the hemithorax. In our study, we recognized that the central location may be associated with lymph node metastasis, but the tumor location seemed to have less impact on a GGO-predominant lesion [27]. Preoperative serum CEA levels are associated with tumor aggressiveness and disease prognosis. CEA is a predictor for mediastinal nodal metastasis in clinical stage IA NSCLC patients [15], and is also associated with a significantly worse 5 year mortality rate. CEA alteration may be influenced by smoking status; in our subgroup analysis of non-smokers, CEA alteration was significantly more frequent in pN+.

Even though lymph dissection has a potentially better survival than lymph nodal sampling, several postoperative complications were treated with lymphadenectomy, including recurrent laryngeal nerve injury, chylothorax, and potentially lethal cardiac tamponade. Considering that the prevalence of lymph nodal metastasis is low in clinically early-stage lung cancer [28], some surgeons prefer nodal sampling instead of complete dissection for a better surgical outcome. On the other hand, in the low-dose CT screening era, there is an increasing number of NSCLC cases that were diagnosed at a very early stage, including adenocarcinoma in situ. In such cases, the necessity of lymphadenectomy remains controversial. Our research helps thoracic surgeons choose a suitable surgical method more easily for limited lymph node dissection or sampling.

We acknowledge that this study has limitations and biases. First, inherent bias associated with retrospective studies could not be avoided by a single surgical team, especially regarding different surgical methods, with those such as sublobar resections or lymph nodal sampling excluded. Second, some missing data, such as the histological subtypes and genetic profile, were not available in some early pathological reports. Third, the extent of lymphadenectomy during the operation depended on the judgment and personal experience of the surgeon. Although PET is a good tool for detecting mediastinal lymphadenopathy, it is not indicated for small lesions of less than 8–10 mm in diameter [29] because of a high rate of false-negative interpretation; additionally, in our country, PET scans are not routinely used for small, peripheral part-solid GGO lesions [30]. Therefore, a thin-section CT scan may be better than PET for lung cancer screening of non-solid nodules. Moreover, a CT scan may help in the detection of early-stage NSCLC and identify the possible inflammatory or infection that causes false positives in PET.

## 5. Conclusions

Our study demonstrated that tumor size, preoperative serum CEA levels, and C/T ratio on CT scan were significant predictive factors for lymph nodal upstaging in lung adenocarcinoma patients with clinical stage T1a-bN0M0. For patients with a tumor size < 1 cm, serum CEA levels < 5 ng/mL, and C/T ratio < 0.5, the avoidance of standard lobectomy or lymph node dissection may be justified, and a less invasive procedure, such as sublobar resection with nodal sampling or even without lymphadenectomy, may be an alternative approach. However, further studies are needed to verify whether our methods could be implemented in clinical settings and have equivalent outcomes when compared with current standards of care.

## Figures and Tables

**Table 1:** Results between patients of adenocarcinoma with and without lymph nodal metastasis.

Variable ^a^	pN+ (*n* = 28)	pN− (*n* = 324)	*p* Value
Age	59.5 (49–67.75)	60 (52–65)	0.828
Gender (female)	17 (60.7)	210 (64.8)	0.684
Smoking	4 (14.3)	47 (14.5)	>0.999
Familial lung cancer	6 (21.7)	64 (19.8)	0.807
Other malignancy	5 (17.9)	33 (10.2)	0.206
CEA (ng/mL)	2.04 (1.28–5.67)	1.54 (1.42–2.48)	0.28
CEA > 5 ng/mL (*n*)	6 (21.7)	12 (3.7)	0.001
Image Findings			
Tumor Site			0.872
Right upper lobe	10 (35.7)	123 (38.0)	
Right middle lobe	5 (17.9)	40 (12.3)	
Right lower lobe	4 (14.3)	63 (19.4)	
Left upper lobe	6 (21.4)	58 (17.9)	
Left lower lobe	3 (10.7)	40 (12.3)	
Tumor Location			0.003
Central	13 (46.4)	64 (19.8)	
Peripheral	15 (53.6)	260 (80.2)	
Total size (cm)	2.10 (1.79–2.69)	1.93 (1.42–2.48)	0.049
0–1	0 (0)	33 (10.2)	0.499
1–2	28 (100)	291 (89.8)	0.499
Solid size (cm)	1.60 (1.40–1.81)	0.85 (0.00–1.38)	<0.001
0–1	0 (0)	181 (55.9)	<0.001
1–2	28 (100)	143 (44.1)	<0.001
C/T ratio	0.75 (0.59–0.87)	0.43 (0.00–0.66)	<0.001
<0.50	3 (10.7)	197 (60.8)	<0.001
≥0.50	25 (89.3)	127 (39.2)	
<0.75	14 (50)	275 (84.9)	<0.001
≥0.75	14 (50)	49 (15.1)	
Pathological features			
Tumor size (cm)	2.2 (1.73–2.5)	1.6 (1.2–2.1)	<0.001
LN op numbers	13.5 (7–20.75)	13 (8–18)	0.852
N1 op numbers	4 (2–7)	4 (2–7)	0.592
N2 op numbers	7 (3–16.5)	7 (4–12)	0.907
LN postive numbers	2 (1–3)	0	<0.001
N1 positive numbers	1 (0–2)	0	<0.001
N2 positive numbers	1 (0–2.75)	0	<0.001
Differentiation			<0.001
Well	0 (0)	107 (33)	
Not well	28 (100)	217 (67)	
VPI	7 (25)	51 (15.7)	0.005
LVI	20 (71.4)	20 (8)	<0.001
Lepidic predominant	0 (0)	31 (9.5)	0.154

^a^: continuous data are shown as median (interquartile range, IQR), and categorical data as number (percentage); pN+/-: patients with or without pathological lymph nodal metastasis; CEA: cryoembryotic antigen. C/T ratio: consolidation/tumor ratio. LN op numbers: numbers of dissected lymph nodes in operation. VPI: visceral pleural involvement. LVI: lymphovascular involvement.

**Table 2:** Results of somatic mutation.

Genetic Mutation ^a^	*n*	pN+	pN−	*p* Value
EGFR	124 ^b^	4 (44.4)	79 (68.7)	0.155
BRAF	109 ^c^	0 (0)	0 (0)	
KRAS	109 ^c^	0 (0)	3 (2.9)	
HER2	109 ^c^	0 (0)	22 (21.8)	
ALK	42 ^d^	1 (25)	1 (2.6)	

^a^: categorical data are shown as number (percentage); ^b^: 9 of pN+ and 115 of pN−; ^c^: 8 of pN+ and 101 of pN−; ^d^: 4 of pN+ and 38 of pN−.

**Table 3:** The risk factors associated with lymph nodal upstaging according to logistic regression analysis.

		Univariate Predictors		Multivariate Predictors	
Independent Variables	*n*	OR (95% CI)	*p* Value	OR (95% CI)	*p* Value
Gender					
Male	125	1.19 (0.54–2.63)	0.664		
Female	227	1.00			
Smoking					
Smoker	51	0.98 (0.33–2.85)	0.975		
Never Smoker	301	1.00			
CEA (ng/dL)					
≧5	18	7.09 (0.43–20.70)	<0.001	6.80 (1.89–24.40)	0.003
<5	334	1.00		1.00	
Total tumor diameter (cm)					
≧1.7	229	2.64 (0.98–7.11)	0.056		
<1.7	123	1.00			
Solid part diameter (cm)					
≧1.3	115	11.6 (4.28–31.43)	<0.001	4.36 (1.39–13.68)	0.012
<1.3	237	1.00		1.00	
C/T ratio					
≧0.50	152	12.93 (3.82–43.70)	<0.001	4.93 (1.16–21.02)	0.031
<0.50	200	1.00		1.00	
C/T ratio					
≧0.75	63	5.61 (2.52–12.50)	<0.001	1.38 (0.53–3.57)	0.51
<0.75	289	1.00		1.00	
Tumor location					
Central	77	3.52 (1.60–7.77)	0.002	4.64 (1.84–11.70)	0.001
Peripheral	275	1.00		1.00	

OR: odds ratio; CI: confidence interval. CEA: cryoembryotic antigen.

## Data Availability

Please contact the corresponding author J.-S.C. (chenjs@ntu.edu.tw).

## Abbreviations

NSCLC	Non-small cell lung cancer
CT	Computed tomography
LND	Lymph node dissection
MRI	Magnetic resonance imaging
PET	Positron emission tomography
GGO	Ground-glass opacity
CEA	Carcinoembryonic antigen
C/T ratio	consolidation-tumor ratio
ROC curve	Receiver operating characteristic curve
IQR	Interquartile range
